# Clinical and molecular characterization of a patient with *MBTPS1* related spondyloepiphyseal dysplasia: Evidence of pathogenicity for a synonymous variant

**DOI:** 10.3389/fped.2022.1056141

**Published:** 2023-01-11

**Authors:** Yeqing Yuan, Qiaoli Zhou, Chunli Wang, Wei Zhou, Wei Gu, Bixia Zheng

**Affiliations:** ^1^ Department of Endocrinology, Children's Hospital of Nanjing Medical University, Nanjing, China; ^2^ Nanjing Key Laboratory of Pediatrics, Children's Hospital of Nanjing Medical University, Nanjing, China

**Keywords:** spondyloepiphyseal dysplasia, whole exome sequencing, MBTPS1, synonymous variant, exon skipping

## Abstract

**Background:**

A novel autosomal recessive skeletal dysplasia resulting from pathogenic variants in membrane-bound transcription factor peptidase, site 1 (*MBTPS1*) has been recently delineated. To date, only three patients have been reported.

**Methods:**

In this study, we reported the clinical and molecular features of a Chinese boy who was diagnosed with spondyloepiphyseal dysplasia. The effects of variants on mRNA splicing were analyzed through transcript analysis *in vivo* and minigene splice assay *in vitro*.

**Results:**

The proband mainly showed short stature, special facial features, cataract, hernias, and serious sleep apnea syndrome. Growth hormone stimulation tests suggested the boy had growth hormone deficiency. Imaging examinations suggested abnormal thoracolumbar vertebrae and severely decreased bone mineral density. Genetic analysis of *MBTPS1* gene revealed two novel heterozygous variants, a nonsense mutation c.2656C > T (p.Q886*, 167) in exon 20 and a synonymous variant c.774C > T (p.A258=) in exon 6. The transcript analysis *in vivo* exhibited that the synonymous variant c.774C > T caused exon 6 skipping. The minigene splice assay *in vitro* confirmed the alteration of *MBTPS1* mRNA splicing and the exon skipping was partially restored by an antisense oligonucleotide (ASO) treatment.

**Conclusion:**

Notably, we report a Chinese rare case of spondyloepiphyseal dysplasia and validate its pathogenic synonymous variant in the *MBTPS1* gene.

## Introduction

Spondyloepiphyseal dysplasia, Kondo-Fu type (SEDKF, OMIM #618392) is a rare autosomal recessive skeletal dysplasia, which is caused by pathogenic variants in *MBTPS1*, initially described by Kondo and Fu in 2018 (1). To date, only three patients have been reported to carry the *MBTPS1* variants. The affected individuals had similar clinical manifestations, presenting severely growth retardation, dysmorphic facial features including large ears, prominent forehead and cheekbones, skeletal dysplasia and cataract (1–3). Besides, epilepsy, craniosynostosis and hernia were reported in different individuals.

Site-1 protease (S1P), encoded by *MBTPS1*, is ubiquitously expressed in the Golgi and proteolytically activates unique membrane-bound latent transcription factors. S1P participate in the metabolism of cholesterol and fatty acid, ER stress response and lysosome biogenesis (4–6). The defective S1P function causes ER retention of collagen in chondrocytes and abnormal secretion of lysosomal enzymes, further leading to apoptosis of chondrocytes and lysosomal enzyme–mediated degradation of the bone matrix. These may explain the importance of S1P for skeletal development in humans and provide a possible mechanism for the association between mutations in the *MBTPS1* gene with the pathogenesis of SEDKF (1).

According to the Human Genome Mutation Database (HGMD), seven mutations have been described in *MBTPS1* gene so far, while synonymous variants have not been reported among them. It was considered that synonymous variants have no influence on gene expression since they did not change the amino acid sequence. However, many advanced findings show that synonymous variants play important roles in RNA transcription and protein translation (7–10), which requires us to perform the necessary functional assays to verify the pathogenicity of these variants.

In this study, we report a Chinese case of SEDKF harboring novel compound heterozygous *MBTPS1* variants including a synonymous variant c.774C > T and demonstrate that this variant is associated with abnormal mRNA processing.

## Materials and methods

### Genetic analysis

Whole exome sequencing (WES) was performed as previously described (PMID: 32153641). In brief, genomic DNA was isolated from blood lymphocytes using the DNA isolation kit (Tiangen, China). Genomic DNA was sheared into fragments and then hybridized with the xGen Exome Research Panel v1.0 probe sequence capture array from IDT (Integrated Device Technology, USA) to enrich the exonic region. The enriched libraries were analyzed on an Illumina HiSeq XTen (Illumina, USA) platform. Variant analysis was performed by geneticists, who had knowledge regarding clinical phenotypes, pedigree structure, genetic mapping, and in line with proposed guidelines. Variants were confirmed by Sanger sequencing and for segregation of phenotype with genotype. All variants were denoted based on the NCBI reference sequence for *MBTPS1* (NM_003791).

### Bioinformatics predictions

To analyze the potential effect of synonymous variant on putative splicing regulatory elements, we used Splice AI, NNSplice, and ESE Finder 3.0 program.

### Transcript analysis *in vivo*

Total RNA was extracted from peripheral leukocytes of the proband and his parents and the normal control leukocyte was extracted from a healthy volunteer blood sample. RNA was isolated with the QIAGEN miRNeasy Mini Kit, and then reverse-transcribed into cDNA by using random hexamers and the SuperScript III transcriptase (Invitrogen). Reverse transcription PCR (RT-PCR) was performed with 100 ng of each cDNA as template. PCR products were separated on 1.5% agarose and sequenced with an ABI 3130 genetic analyzer (Applied Biosystems).

### Antisense oligonucleotides design

Antisense oligonucleotides were designed as described previously (PMID: 29188506). Briefly, the RNA structure nearby the variant c.774C > T of *MBTPS1* gene was analyzed with the Mfold software [RNAfold web server (http://rna.tbi.univie.ac.at/cgi-bin/RNAWebSuite/RNAfold.cgi)]. Then, we designed an ASO sequence with the length of 25 nucleotides and had a Tm above 48°C and a GC content between 40% and 60%. The exact sequences are ACACGAUGCAGUGAGGGAAAAAAG (ASO1). The ASO was synthesized with phosphorothioate backbone and a 2-O-methyl sugar modification (Tsingke Biological Technology, China).

### Minigene assay *in vitro*

We confirmed the splicing effect of the synonymous variant c.774C > T using a pSPL3 minigene reporter vector as previously described (PMID: 29637721). In brief, genomic fragments containing the variants located in exon 6 were amplified by PCR from the patients' genomic DNA using primers (MBTPS1-EXON6: Forward accagaattctggagctcgagACGTCTAAGGGGATCGTAGA; Reverse 5′-atcaccagatatctgggatccTGCAGTATGAATGGCTCAGC-3′). Then, the PCR products were cloned into pSPL3 vector using the ClonExpressTM II One step Cloning Kit (Vazyme Biotech Co., Ltd). All constructs were sequenced by Sanger sequencing. HEK293 cells were cultured in 12-well plates and transfected with 1 µg purified pSPL3, wild-type and variant constructs plasmids using Lip2000 (Invitrogen). After overnight incubation, cells were transfected with ASO at the concentration of 0.5 μM. Forty-eight hours after ASO delivery, cells were harvested for transcriptional analysis by RT-PCR. Finally, aberrant splicing transcripts amplification by RT-PCR, PCR product separation by agarose gel and proven by Sanger sequencing were performed.

## Results

### Clinical presentation

**Figure 1:**
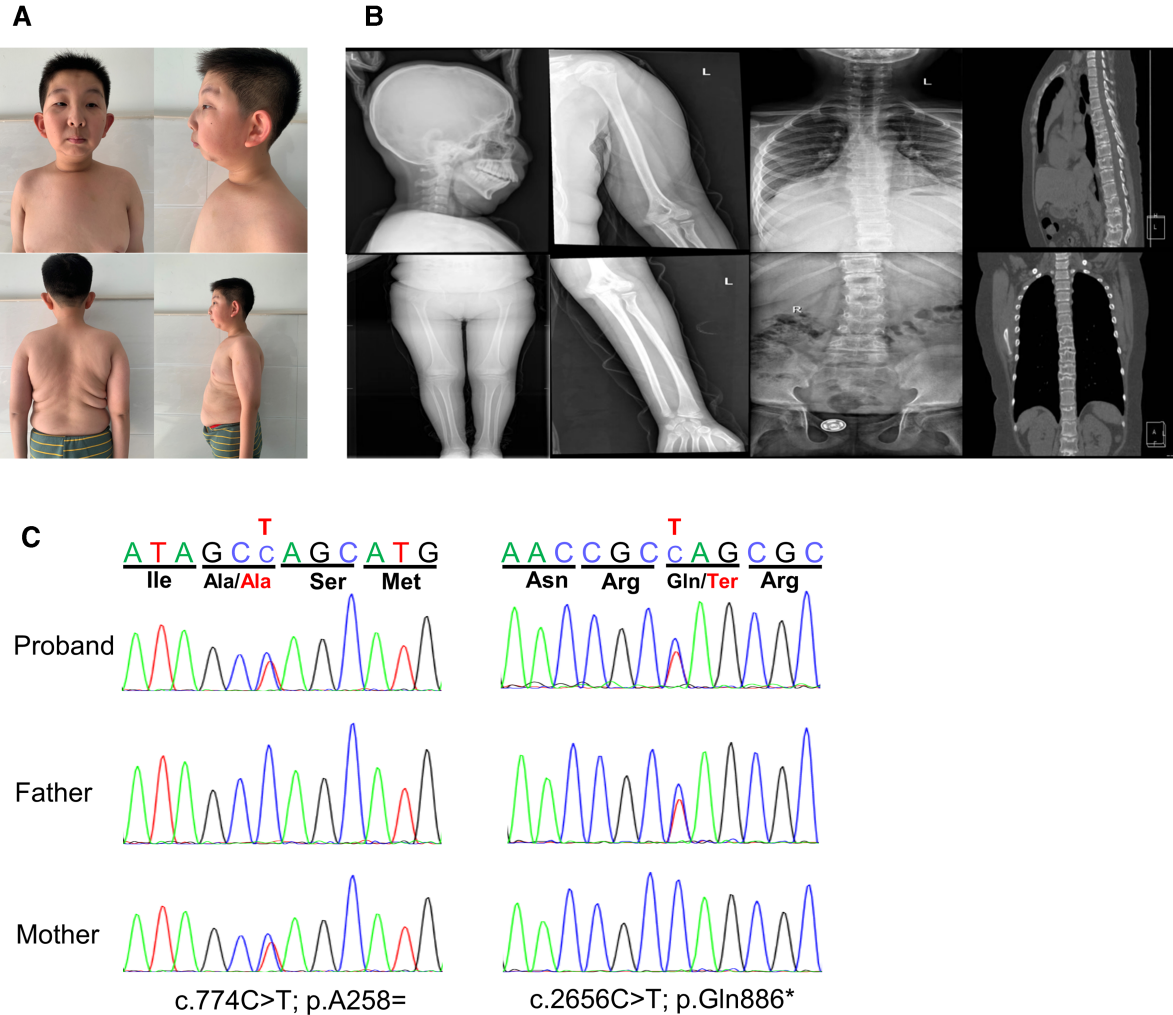
Clinical features and gene variants of the proband. (**A**) The patient exhibited dysmorphic facial features with high nose bridge, epicanthus, prominent cheekbones, retromicrognathia, big ears. Other features included pectus carinatum, scoliosis, large and protruding abdomen. (**B**) Radiographies showed multiple thoracolumbar vertebral bones dysplasia with scoliosis, the rough edges of vertebra, endplate bone defects, and the narrow intervertebral space, while no obvious abnormalities were seen in the long bones of limbs and the skull. (**C**) The compound heterozygous variants in the *MBTPS1* gene detected in our patient. The two variants, c.774(exon6)C > T and c.2656(exon20)C > T, were inherited from her mother and father, respectively.

The proband was born as the first child to healthy, non-consanguineous parents. He was delivered *via* cesarean section at full-term gestation because of the twisting of the umbilical cord around the fetal neck. His birth weight and height were 2.8 kg and 50 cm, respectively. At 2 years of age, he underwent a surgery for bilateral cataracts. At the age of 5 years, he underwent high ligation for bilateral inguinal hernias, followed by surgery for left-sided cryptorchidism at 11 years of age. The patient thereafter was admitted to our department due to postnatal growth retardation at the age of 12 years. On physical examination, his height was 137.4 cm [−2.5 standard deviation score (SDS)] and weight was 40 kg (−0.68 SDS). He was noted to have facial dysmorphic features including high nose bridge, epicanthus, prominent cheekbones, retromicrognathia and big ears (Figure 1A). Moreover, he presented with a pectus carinatum, hyperextended fingers and obvious accumulation of fat on the chest and abdomen (Figure 1A). External genitalia showed pubertal Tanner stage I (penile length of 3 cm, testicular volume of 2 ml). He walked with a staggering gait, but there was no significant decrease in muscle strength and muscle tone.

Laboratory tests revealed that blood cell count, blood sugar, HbA1c, insulin, liver and renal function were normal. Blood lipids evaluation showed decreased high-density lipoprotein [0.56 mmol/L, normal range (NR) 1.16∼1.42 mmol/L], apolipoprotein B (0.58 mmol/L, NR 0.66∼1.33 mmol/L) and apolipoprotein A1 (0.7 mmol/L, NR 1.04 ∼2.02 mmol/L), whereas the very long-chain fatty acids were in the normal range. Organic acids analysis with gas chromatography-mass spectrometryin urine and acylcarnitines analysis with liquid chromatography-tandem mass spectrometryin dried blood spot was normal. The results of blood gas, ammonia, lactate, coagulation function and tumor indicators did not reveal abnormal values. The main bone metabolism indicators including serum calcium, phosphorus, other electrolytes, alkaline phosphatase and parathyroid hormone were normal, while vitamin D was low (32.727 nmol/L, NR:>75 nmol/L). Thyroid and adrenocortical functions were normal. The serum level of IGF-1 and IGFBP-3 were normal. The peak values of growth hormone were 0.548 ng/ml with arginine and 0.320 ng/ml with clonidine, respectively, in the growth hormone stimulation test. His bone age (13 years) was equivalent to his chronological age. Wechsler Intelligence Scale for Children Test revealed mild intellectual disability (IQ = 57). The bone mineral density was severely decreased with a Z-score of −2.9. Radiographic examination manifested multiple thoracolumbar vertebral bones dysplasia with scoliosis. The computed tomography (CT) scan showed pectus carinatum, the rough edges of vertebra, endplate bone defects, and the narrow intervertebral space, while no obvious abnormalities were seen in the long bones of the limbs and the skull (Figure 1B). Abdominal ultrasound, echocardiogram and MRI of pituitary were normal. The patient showed severe obstructive sleep apnea with apnea hypopnea index of 46.4 (NR ≥ 5) in polysomnography, without adenotonsillar hypertrophy (Figure 1B).

### Genetic findings

Analysis of WES data identified compound heterozygous variants in the *MBTPS1* gene: c.2656C > T (p.Q886*) and c.774C > T (p.A258=), which were inherited from his father and mother, respectively (Figure 1C). The nonsense variant c.2656C > T (p.Gln886Ter, 167) in exon 20 was predicted to generate a premature termination codon resulting in truncated proteins. The other variant c.774C > T (p.Ala258Ala) in exon 6 was predicted to be a synonymous variant. These two variants were not found in the Single Nucleotide Polymorphism Database (dbSNP), the 1,000 Genomes Project Database or the Genome Aggregation Database (gnomAD). According to the American College of Medical Genetics and Genomics (ACMG) guidelines (11), the variant c.2656C > T (p.Q886*) could be classified as likely pathogenic (PVS1 + PM2) and the variant c.774C > T (p.A258=) could be uncertain significance (PM2 + PM3).

### Bioinformatics prediction

**Figure 2:**
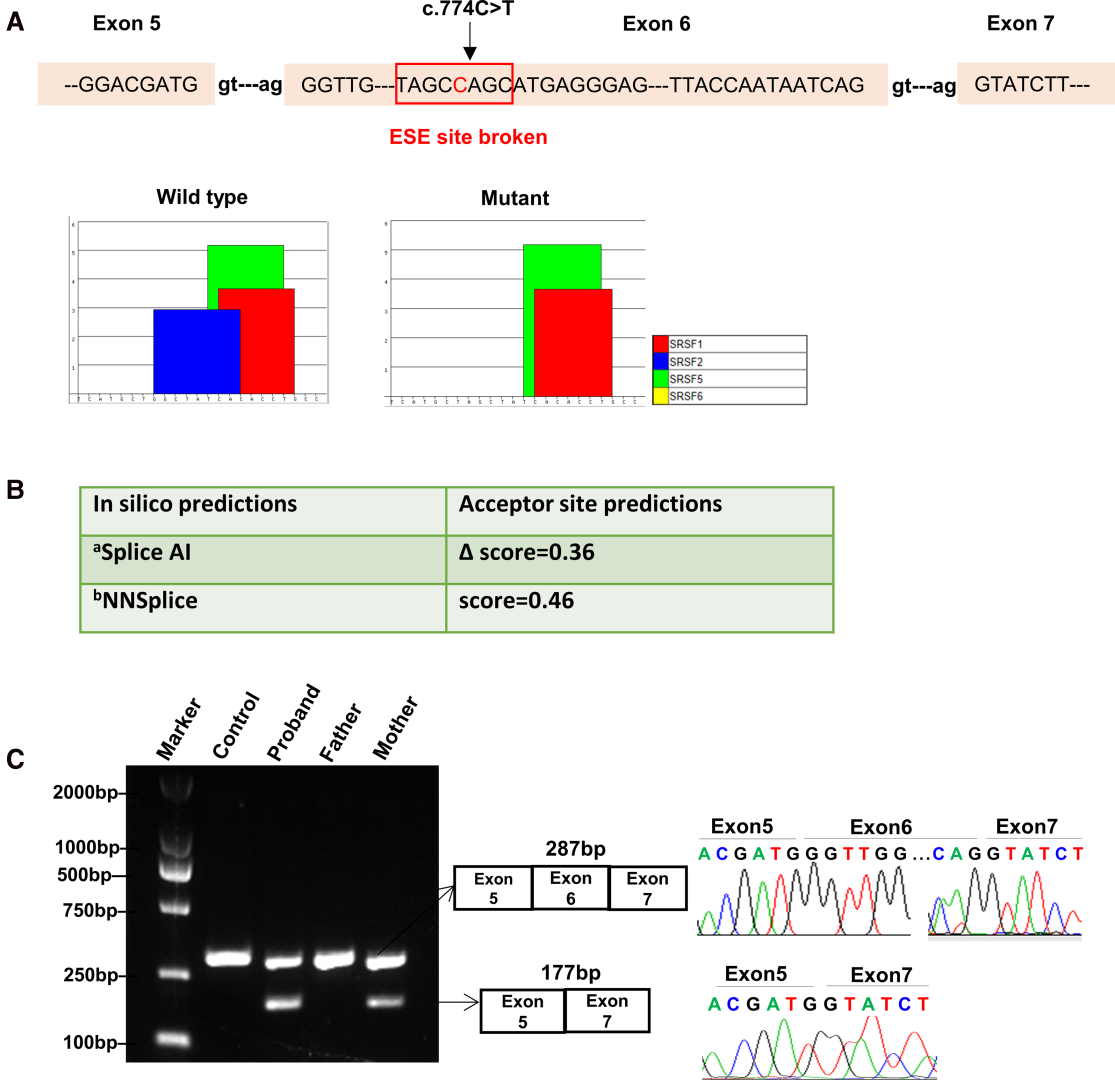
Bioinformatics predictions and transcript analysis *in vivo* for the synonymous variant c.774C > T in the *MBTPS1* gene. (**A**) ESE Finder 3.0 program predicted that c.774C > T might affect auxiliary cis-acting splicing regulatory elements by destroying the original exonic splicing enhancer (ESE) binding to SRSF2. (**B**) In silico prediction results for c.774C > T using Splice AI and NNSplice. a, Delta score of a variant can be interpreted as the probability of the variant being splice-altering: 0.2 (high recall), 0.5 (recommended), and 0.8 (high precision) cutoffs.; b, Splice site predictions for acceptor score cutoff 0.40. (**C**) Gel electrophoresis of RT-PCR fragments *in vivo* showed that c.774C > T caused abnormal mRNA splicing leading to two transcripts differed by about 100 bp. Sequence analysis of the patient and mother's mRNA derived RT-PCR products showed that the shorter transcript lacked a sequence corresponding to exon 6 of the *MBTPS1* gene.

Synonymous variants are challenging to interpret. Whilst it is widely acknowledged that synonymous variants may sometimes contribute to disease phenotypes by altering splicing machinery which ultimately requires experimental validation (8, 12). We firstly used Splice AI and NNSplice to analyze the potential effects of the synonymous variant c.774C > T on mRNA splicing. As a result, they outputted moderated scores for accept site loss (Figure 2B). While ESE Finder 3.0 program showed this variant might affect auxiliary cis-acting splicing regulatory elements by destroying the original ESE binding to SRSF2, ultimately leading to abnormal splicing (Figure 2A).

### Splicing assay *in vivo*

In order to validate the prediction *in silico*, RNA was isolated from the patient and his mother's peripheral white blood cells. cDNA fragments covering the coding region of the c.774C > T variant were obtained by RT-polymerase chain reaction (RT-PCR) and sequenced.

The results showed that the proband and mother's leukocytes expressed two transcripts that differed by about 100 bp. Sequence analysis of the products showed that the shorter transcript lacked a sequence corresponding to exon 6 (Figure 2C). Taken together, we rated the variant c.774C > T as pathogenic based on the ACMG guidelines. These two variants were submitted to the LOVD database.

### In vitro minigene splicing assay and ASO block

**Figure 3:**
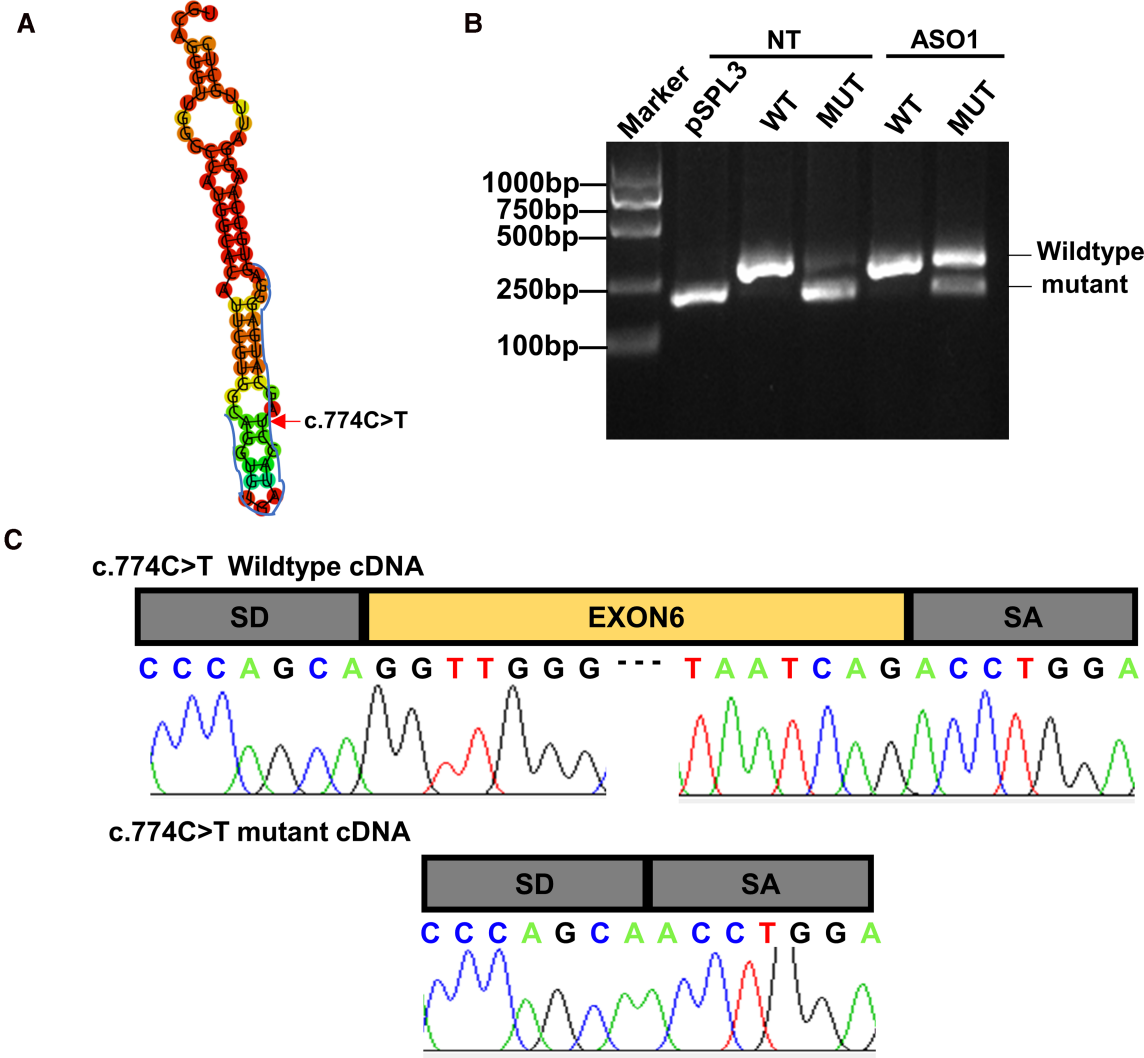
Antisense oligonucleotide partially rescue the exon skipping caused by c.774C > T variant in the *MBTPS1* gene in HEK293 cells. (**A**) Positions of the antisense oligonucleotides (ASO) for the c.774C > T variant in this study. (**B,C**) Analysis of splicing correction by RT-PCR upon ASO. Wild-type(WT) minigene and the corresponding mutant(MUT) minigene containing the variant c.774C > T were transfected in HEK293. The ASO were then delivered except the non-treated lanes(NT). The exon skipping caused by c.774C > T was efficiently rescued by ASO.

The minigene splicing assay confirmed that the synonymous variant c.774C > T could cause exon 6 skipping compared with the wild type (Figure 3B). Then, the abnormal mRNA splicing was confirmed by Sanger sequencing (Figure 3C). After elucidating the pre-mRNA splicing defect of variant c.774C > T, we aimed to design a therapeutic approach to block the impact of the variant on auxiliary splicing sequences (Figure 3A). The effect of the ASO at the RNA level was assessed by RT-PCR. As shown in Figure 3, the ASO partially restored the exon skipping (Figure 3).

## Discussion

So far, only three *MBTPS1*-related SEDKF cases were reported (1–3). They shared the common clinical manifestations with our patient: short stature related to skeletal dysplasia, distinctive facies with large ears, high cheekbones, retromicrognathia, concomitant symptoms like cataracts, hernia and so on. Besides, our patient showed severe obstructive sleep apnea syndrome (Table 1).

**Table 1:** Summary of the clinical and genetic features of spondyloepiphyseal dysplasia with *MBTPS1* mutation reported in the literature vs. our proband.

Age	11.5 years	An adult	5 years	12 years
Country	USA	Germany	Brazil	China
Gender	female	male	female	male
History of birth	At term, 2.07 kg	At term, 2.4 kg	Preterm, 1.97 kg	At term, 2.8 kg
Variant	c.285dupT (p.D96X)^P^ c.1094A > G (p.D365G)^M^	c.1094A > G (p.D365G)^PM^	c.2948G > A (p.W983*)^PM^	c.2656C > T (p.Q886*)^P^ c.774C > T (p.A258=)^M^
Development				
motor milestones	delayed	delayed	delayed	N
speech	N	+	+	N
intellectual disability	N	+	+	+
Facial features				
prominent forehead	+	N	+	N
prominent cheekbones	+		+	+
large ears	+	+	+	+
retromicrognathia			+	+
thick lips			+	N
Skeletal dysplasia				
short stature	+	+	+	+
pectus carinatum	+	+	+	+
kyphosis	+	+	+	N
waddling gait	+			+
Hernia	+	+	N	+
Cataract	+	+	+	+
Protruding abdomen		+	+	+
Epilepsy			+	N
Obstructive sleep apnea				+
Biochemical tests				
liver function			increased	N
renal function			N	N
blood lipids	lower HDL		N	lower HDL
organic acids analysis			N	N
Bone indicators				
serum calcium	N		N	N
serum phosphorus			N	N
vitamin D	N		N	decreased
alkaline phosphatase	N		N	N
parathyroid hormone	N		N	N
Lysosomal enzymes	elevated	elevated	elevated	
Echocardiogram	N		N	N
Abdominal ultrasound			hepatomegaly	N
Skeletal radiographs				
skull			+	N
long bones	+		+	N
epiphyses	+		+	+
vertebrae	+		+	+
Osteopenia	+		+	+
References	1	2	3	our proband

N, normal or no; blank, no report; P, from paternal variant; M, from maternal variant; HDL, high-density lipoprotein.

As a new-recognized rare type of skeletal dysplasia, SEDKF has not been updated into the newest version of the Nosology and Classification of Genetic Skeletal Disorders (13). According to the radiological tests, we could identify the distinctive skeletal changes from our proband such as flattened thoracolumbar vertebral bodies, the rough edges of vertebra, endplate bone defects, and the narrow intervertebral space. These radiological findings helped to distinguish *MBTPS1*-related SEDKF from other very rare skeletal disorders, like Spondylocostal dysostosis 6 which showed complex vertebral segmentation defects in the cervico-thoracic spine (14) and MYH3-related arthrogryposis which displayed unilateral carpal bone fusion and multiple vertebral fusions (15). Moreover, all of them had their own distinctive facial features and responsible genes.

A broader conceptual framework that the skeletal growth plate is responsible for linear growth and short stature is caused by growth plate dysfunction is formulated now (16). Chondrogenesis at the skeletal growth plate results in the childhood linear growth and the cartilaginous growth plate consists of linear columns of differentiated chondrocytes and extracellular matrix (17–19). The *MBTPS1* gene encodes S1P, which plays an important role in ER retention of collagen in chondrocytes, apoptosis of chondrocytes and degradation of the bone matrix (1). These might explain the relationship between the mutated *MBTPS1* with our patient's clinical features especially the skeletal dysplasia characterized by hyperextension of the palms, pectus carinatum scoliosis, multiple thoracolumbar vertebral bones dysplasia and severely decreased bone density.

There are only a few reports of other clinical phenotypes related to *MBTPS1*. One study described an adult proband with a novel heterozygous *de novo* mutation in *MBTPS1* showed episodic hyperCKemia and focal myoedema (20). Another research introduced two unrelated and ethnically diverse children probands presented a new entity named cataract, alopecia, oral mucosal disorder, and psoriasis-like (CAOP) syndrome and WES and Sanger sequencing of both patients identified compound heterozygous variants in the *MBTPS1* gene (21). Two phenotypes markedly different from our proband could be due to gain-of-function or the different involved organelles. The polymorphism of the genotype and variability of the phenotype make it necessary to carry on genetic analysis and functional tests to elucidate the genotype-phenotype relationship more accurately.

It had been validated that a nucleotide duplication (c.285dupT) in *MBTPS1* exon 3 could create a nonsense change in which mutant transcript encoded an S1P that lacked the entire catalytic domain. In addition, a nucleotide substitution (c.1094A > G) in exon 9 resulted in a missense variant and created a dominant splice donor site in exon 9, leading to an alternatively spliced transcript with a 41-bp deletion of exon 9. And nonsense-mediated mRNA decay (NMD) inhibitor (cycloheximide) could increase these mutant *MBTPS1* transcripts, indicating that mutant transcripts were unstable due to the NMD quality control system (1). To date, no synonymous variants in the *MBTPS1* gene have been reported. Owing to the belief that the structure of proteins is determined by changes in the sequence of amino acids, synonymous variants are generally considered to be “silent”, so their pathogenicity requires functionally validated. In our study, bioinformatic prediction tools Splice AI and NNSplice outputted moderated scores for accept site loss at first, while ESE Finder 3.0 program suggested that the synonymous variant c.774C > T disrupted exonic splicing regulatory sequences, destroyed the original ESE, which was predicted to regulate alternative splicing, leading to abnormal exon skipping. The exon-skipping event produced a truncated mRNA, which was expected to result in a frameshifted MBTPS1 protein or downstream premature termination codon that may initiate the NMD process. Therefore, it is more importantly and necessarily to conduct functional studies than to perform bioinformatics analysis merely to verify the pathogenicity of synonymous variants.

Unfortunately, we have not yet found a certain treatment for the disease. Growth hormone stimulation tests suggested our patient had growth hormone deficiency, but according to the study of Kondo et al., the patient received growth hormone therapy for 1 year appeared limited response (1). In addition, S1P-deficient short stature might be compared with other skeletal disorders which tend to respond inefficiently to rGH (22, 23). Altogether, the efficiency of rGH to the SEDKF remains doubtful. Obstructive sleep apnea syndrome has a large impact on cognitive function in children than in adults by acting on the plastic brain structures which can change the neuro-psychic development, learning skills, and social interactions (24, 25). It suggests that our patient may require necessary intervention such as CPAP therapy, surgical treatment, drugs and orthodontic appliances. Skeletal deformities such as pectus carinatum and scoliosis may need evaluation from an orthopedic specialist. Mechanism research operated by Kondo et al. revealed potential individualized therapies such as ASO and sodium phenylbutyrate may have a certain therapeutic effect on the disease, but there is still lack of corresponding clinical trials (1). Likewise, our study demonstrated that ASO could partially restore the exon skipping, which not only validate the disease-causing variants of *MBTPS1*, but also provide a promising gene therapy approach for this disease.

## Conclusions

We reported the first case of *MBTPS1* gene-related SEDKF in China caused by two novel pathogenic variants, and validated that the synonymous variant is associated with the disease through pre-mRNA splicing defect. More importantly, our findings expand the clinical spectrum of this disease and underline the importance and necessity of functional testing for synonymous variants especially those involving uncertain significance.

## Data Availability

The raw data supporting the conclusions of this article will be made available by the authors, without undue reservation.
